# Comparative evaluation of ten blood biomarkers of inflammation in regular heated tobacco users and non-smoking healthy males–a pilot study

**DOI:** 10.1038/s41598-024-59321-y

**Published:** 2024-04-16

**Authors:** Beata Świątkowska, Mateusz Jankowski, Dorota Kaleta

**Affiliations:** 1 Department of Hygiene and Epidemiology, Medical University of Lodz, Żeligowskiego 7/9 Street, 90-752 Łódź, Poland; 2 Department of Population Health, School of Public Health, Centre of Postgraduate Medical Education, Kleczewska 61/63 Street, 01-826 Warsaw, Poland

**Keywords:** Biomarkers, Epidemiology

## Abstract

Heated tobacco products (HTPs) are novel tobacco products that are alternatives to cigarettes. The study aimed to investigate the effect of HTPs on blood biomarkers of inflammation as well as to provide a comparative evaluation between daily heated tobacco users and healthy men who do not use nicotine products. This case–control study was carried out among 92 healthy males in Poland (Lodz-Province) aged 20–56 years: 44 daily heated tobacco users (daily use in the past 90 days) and 48 controls who do not use nicotine products. The history of use of the nicotine-containing products was self-reported and verified using a saliva cotinine test. A 20 ml blood sample was collected and the levels of ten blood biomarkers were analyzed. Among all heated tobacco users (n = 44), only the levels of interleukin 8 (IL-8) were significantly higher when compared to controls: 6.86 vs. 3.95 (p = 0.01). Among exclusive heated tobacco users (n = 33), the levels of IL-8 were also significantly higher when compared to controls: 7.76 vs. 3.95 (p = 0.01). IL-8 level was positively correlated (r = 0.37; p = 0.01) with the daily number of heated tobacco sticks. Out of 10 different biomarkers of inflammation, only IL-8 levels were significantly elevated in heated tobacco use compared to controls.

## Introduction

Heated tobacco products (HTPs) are novel tobacco products that are alternatives to cigarettes and combustible tobacco products^1–3^. HTPs operate based on an electronically controlled heating element that heats dedicated tobacco-containing sticks up to 350 °C and generates aerosol inhaled by the user^3^. Nicotine levels in aerosol generated by HTPs are estimated at 70–80% as those generated by combustible cigarettes^4^. When compared to electronic cigarettes (e-cigarettes), HTPs-dedicated sticks contain processed tobacco which is a source of nicotine, instead of a dedicated nicotine-containing liquid like in e-cigarettes^5^.

HTPs have been marketed since 2014^3^. The market is dominated by the heating tobacco devices of major tobacco companies, including “IQOS” from Philip Morris International, “glo” from British American Tobacco, and “Ploom TECH” from Japan Tobacco^3^. It is estimated that 5% of the global population has ever used HTPs and 1.5% of the global population has been identified as current HTP users^6^. In recent years, the prevalence of HTPs increased rapidly. In Japan, the prevalence of HTPs increased from 0.2% in 2015 to 11.3% in 2019^7^. In Poland, the prevalence of daily HTP use increased from 0.4% in 2019 to 4.0% in 2022^8^. The global prevalence of HTPs is estimated to rise in the coming years^6^.

With increasing public awareness of HTPs and the growing number of users, questions about the health effects of HTP use are increasing^2,9,10^. HTPs are being marketed as reduced exposure alternatives to combustible cigarettes^3,9,11^. Most of the scientific evidence on the health effects of HTP use is based on laboratory evaluation of aerosol generated by HTP use as well as experimental studies on animal and cellular models, with limited data from human-based studies^3,9^. Both industry-funded studies and independent research confirmed that the concentration of chemical compounds in aerosol generated during HTP use is lower than during smoking combustible cigarettes^3,9,12,13^. In general, toxic compounds related to combustion process were significantly reduced, but heated tobacco products are not risk-free^3,9,12^. Findings from the experimental animal and cellular studies showed that aerosol from HTPs has lower toxicity than cigarette smoke^3,9^. There are concerns that while it may expose users to less of some toxins that are also found in traditional cigarettes, the use of heated tobacco exposes users to other toxic substances whose toxicological profiles of short- and long-term health effects are unknown^2,3,9,10^. Nevertheless, HTP use may induce oxidative stress, and inflammation and increase the risk of respiratory tract infections^9,14^. While most research funded by the tobacco industry has demonstrated the health advantages of switching from traditional cigarettes to HTP, certain independent studies point to some possible negative effects of HTP aerosol exposure^3,9,10^.

There is a limited number of human-based studies on the health effects of HTP use, mostly on short-term health effects^15–17^. Majek et al. showed that the use of HTP for 5 min evoked an increase in heart rate and blood pressure as well as a decrease in fractional exhaled nitric oxide (FeNO) levels^15^. Goebel et al. reported small airway obstruction and resistance as well as nicotine-related acute increase in arterial stiffness and cardiovascular stress after HTP use^16^. Lyytinen et al. reported increased arterial stiffness and platelet thrombus formation 5 min after the HTP use in a group of healthy young adults^17^.

Smoking-induced oxidative stress, chronic inflammation, and endothelial dysfunction are considered to play major roles in the pathogenesis of tobacco-related diseases^3,9,15–17^. However, little is known about blood-based biomarkers of inflammation in regular heated tobacco users.

The study aimed to investigate the effect of heated tobacco products on blood biomarkers of inflammation as well as to provide a comparative evaluation between daily heated tobacco users and healthy men who do not use nicotine products.

## Material and methods

### Study design and population

This case–control study was carried out among 92 healthy males in Poland (Lodz Province): 44 daily heated tobacco users (past 90 days) and 48 controls who do not use nicotine products. All procedures were carried out in a medical facility and supervised by a physician. Subjects were recruited using an active recruitment network, using the Medical University of Lodz and a network of private medical facilities operating in the Lodz Province. Volunteers who declared a conscious willingness to participate in the study were screened to assess the inclusion and exclusion criteria and qualifications to participate in the study. Subjects were asked to undergo the examination without a meal, and heated tobacco users were asked not to use nicotine products on the day of the tests. A physical examination and a short interview were performed to assess the current health status. The participants were then asked to fill out a set of questionnaires and blood samples were collected. All participants provided written informed consent. The study protocol was reviewed and approved by the Ethics Committee of the Medical University of Łódź (decision number: RNN/235/22/KE of 8/11/2022). All procedures performed in this study were in accordance with the ethical standards of the institutional research committee and with the Declaration of Helsinki.

### Inclusion and exclusion criteria

#### Heated tobacco users

Males aged 20–56, without chronic diseases, who declare regular use of HTPs (IQOS or glo) – at least one heated tobacco stick daily in the last 90 days. Among heated tobacco users, subjects who use concurrently use e-cigarettes regularly (at least 15 days/month) or concurrently use combustible cigarettes/factory made cigarettes were classified into a subgroup of dual users (subgroup 1: HTPs + e-cigarette; subgroup 2: HTPs + cigarette).

#### Controls

Males aged , 20–56, without chronic diseases, who do not use nicotine products and did not smokers or have smoked less than 100 cigarettes in their life who have not used nicotine replacement therapy in the last 90 days, and who have used an e-cigarette no more than once in their entire life and e-cigarette use has occurred more than 90 days before the examination, and have used HTP no more than once in their entire life and HTP use has occurred more than 90 days before the examination. The control group was recruited in such a way that it corresponded to the demographic characteristics of the group of heated tobacco users.

### Smoking status

The history of use of the nicotine-containing products was based on self-reported and verified using a saliva cotinine test (Salimetrics 1-2002, Stratech Scientific Ltd, UK; cotinine concentration in ng per ml). The mean cotinine concentration was 0.55 ng/ml among control, which is below the cut-of value for non-smokers published on the website^18^ of the test manufacturer (5 ng/ml), comparted to 360.9 ng/ml (p < 0.001) among HTPs (with cut-off value for smokers 100 ng/ml)^18^.

Participants were asked about ever and past 90 days use of heated tobacco use, e-cigarette use, and cigarette smoking. Those who declared the use of nicotine-containing products (current or in the past) were asked about the characteristics of nicotine product use (over 20 questions on each type of nicotine-containing product). A total of 88 questions on nicotine-containing products were addressed. Heated tobacco users were asked about the frequency of use, the number of heated tobacco sticks consumed per day, the type of heated tobacco device used, heated tobacco stick, motivation for HTPs use, indoor use of HTPs, harm perception of HTPs, and self-reported health effects of HTPs use, as well as the addictive potential of HTPs. Questions on the history of HTPs use were based on the Global Adults Tobacco Survey (GATS) and adapted to HTPs use^19^.

### Measures

A 20 ml blood sample was obtained from each subject. Levels of the following blood-based biomarkers of inflammation^20^ in blood serum were analyzed:

- Proinflammatory cytokine levels: interleukin (IL) IL-1β, IL-6, IL-8, IL-10, IL-12p70, tumor necrosis factor alpha (TNF-α) (Magnetic Luminex^®^ performance assay, FCSTM09, Luminex Corp, Austin, TX, US). Cytokine levels were presented in picograms per milliliter [pg/ml].
- c-reactive protein (CRP) and fibrinogen levels (MILLIPLEX^®^ human cardiovascular disease (CVD) magnetic bead panel, HCVD3MAG-67 K, Merck, Darmstadt Germany). CRP levels were presented in milligrams per liter [mg/l] and fibrinogen levels were presented in milligrams per deciliter [mg/dl].
- Adhesion molecules: vascular cell adhesion molecule (VCAM-1) and intercellular adhesion molecule (ICAM-1) (Human Luminex^®^ Discovery Assay, LXSAHM-08, Luminex Corp, Austin, TX, US). Adhesion molecule levels were presented in nanograms per milliliter [ng/ml].

All laboratory tests were carried out in the Central Scientific Laboratory of the Medical University of Lodz (CoreLab). All diagnostics procedures were performed by qualified medical personnel with experience in medical diagnostics following the laboratory in accordance with the recommendations of the diagnostic test manufacturers.

### Statistical analysis

Data analysis was performed using procedures available in IBM SPSS Statistics 29 (IBM, Armonk, NY, USA). The normality of distributions was tested using the Shapiro–Wilk test. Differences in the distribution of quantitative variables between the HTPs users and control group were analyzed using Student’s t-test or non-parametric tests *U* Mann–Whitney test. Spearman’s rank correlation was used to analyze the correlation between the blood biomarker levels and the daily number of heated tobacco sticks used in the last 90 days. Data were presented separately for all heated tobacco users (only HTPs or dual use HPTs and cigarette or e-cigarette) and exclusive heated tobacco users (only HTPs in the last 90 days). Statistical significance was assessed at p < 0.05.

## Results

### Characteristics of the study population

A total of 92 healthy males were recruited. The mean age of HTP users was 33.6 years and control 33.4 years (Table 1). Most of the subjects had higher education: (56.8% of HTP users and 60.4% of controls) and lived in cities above 500,000 residents (61.4% of HTP users and 83.3% of controls). Most of the subjects had full-time jobs (59.1% of HTPs users and 64.6% of controls). Among heated tobacco users, 95.5% had ever tried a cigarette and 84.1% had ever tried an e-cigarette (Table 1). Among controls, 18.8% had ever tried a cigarette, 20.8% had ever tried an e-cigarette and 12.5% had ever tried heated tobacco products. Detailed characteristics is presented in Table 1.

**Table 1:** Characteristics of the study population.

	HTPs users (n = 44)	Control group (n = 48)
	n (%)	n (%)
Gender		
Male	44 (100.0)	48 (100.0)
Age		
Mean ± SD	33.6 ± 7.27	33.4 ± 10.20
Min–max	21–46	20–56
Having higher education		
No	19 (43.2)	19 (39.6)
Yes	25 (56.8)	29 (60.4)
Place of residence		
City up to 10,000 residents	6 (13.6)	1 (2.1)
City from 10,000 to 20,000 residents	1 (2.3)	3 (6.3)
City from 20,000 to 50,000 residents	7 (15.9)	2 (4.2)
City from 50,000 to 100,000 residents	2 (4.5)	2 (4.2)
City from 100,000 to 200,000 residents	1 (2.3)	0 (0.0)
City from 200,000 to 500,000 residents	0 (0.0)	0 (0.0)
City above 500,000 residents	27 (61.4)	40 (83.3)
Occupational status		
Full-time job	26 (59.1)	31 (64.6)
Part-time job	5 (11.4)	6 (12.5)
Self-employment	13 (29.5)	7 (14.6)
Student	3 (6.8)	12 (25.0)
Unemployed	1 (2.3)	1 (2.1)
Marital status		
Single	13 (29.5)	14 (29.2)
Married	23 (52.3)	20 (41.7)
Informal relationship	8 (18.2)	14 (29.2)
Ever cigarette smoking	42 (95.5)	9 (18.8)
Ever e-cigarette use	37 (84.1)	10 (20.8)
Ever heated tobacco use	44 (100.0)	6 (12.5)

### Patterns of heated tobacco product use

Out of all heated tobacco product users (n = 44), 11.4% were daily smokers and 15.9% declared daily use of e-cigarettes. In total, there were 10 dual users (HTPs and cigarette or e-cigarette) and 1 triple user (HTPs, cigarette, e-cigarette). Most of the HTP users consumed from 11 to 20 heated tobacco sticks per day (Table 2). The most popular heated tobacco device was IQOS, used by 52.3% of heated tobacco users, 40.9% used glo and 6.8% used lil SOLID 2.0. Mint or menthol flavors were the most common ones, 13.6% used blueberry flavor and only one-half of HTP users used tobacco flavor sticks. Most of the HTPs users used heated tobacco devices indoors (93.2%) and 54.5% used HTPs in places where smoking is prohibited (Table 2). Detailed characteristics of the heated tobacco users is presented in Table 2. The average number of heated tobacco stick packs per week was 5.1 ± 2.9, median of 5 packs per week. Most of the participants used HTP for 3 years (29.5%), one-fifth used HTP for 2 years, 15.9% used HTP for less than 1 year, 13.3% for 4 years and 11.4% of HTP users used heated tobacco products for 5 years (Table 2).

**Table 2:** Characteristics of the heated tobacco users (n = 44).

	HTPs users (n = 44)
	n (%)
Current cigarette smoking	5 (11.4)
Current e-cigarette use	7 (15.9)
Number of heated tobacco sticks used per day	
1	2 (4.5)
2–5	8 (18.2)
6–10	7 (15.9)
11–20	21 (47.7)
Over 20	6 (13.6)
How long do you use heated tobacco products?	
Less than 1 year	7 (15.9)
For 1 year	4 (9.1)
For 2 years	9 (20.5)
For 3 years	13 (29.5)
For 4 years	6 (13.6)
For 5 years	5 (11.4)
Type of heated tobacco device	
IQOS	23 (52.3)
Glo	18 (40.9)
lil SOLID 2.0	3 (6.8)
Most frequently used flavors of tobacco sticks in the last 90 days	
Tobacco flavor	11 (25.0)
Mint flavor	13 (29.5)
Menthol flavor	11 (25.0)
Blueberry flavor	6 (13.6)
Do you use HTPs in places where smoking is prohibited?	
Yes	24 (54.5)
No	20 (45.5)
Do you use HTPs indoors?	
Yes	41 (93.2)
No	3 (6.8)
How soon after waking up do you use heated tobacco?	
Up to 5 min	3 (6.8)
6–30 min	13 (29.5)
31–60 min	12 (27.3)
Over 60 min	16 (36.4)
Do you have difficulty refraining from using heated tobacco in places where it is prohibited?	
Yes	7 (15.9)
No	37 (84.1)
Which heated tobacco stick is the most difficult for you to give up?	
From first in the morning	24 (54.5)
From any other	20 (45.5)
At what time of day do you use heated tobacco more often?	
Within the first hours after waking up	12 (27.3)
During the rest of the day	32 (72.7)

### Blood biomarkers of inflammation levels

A comparative evaluation of blood biomarkers in regular heated tobacco users and healthy males who do not use nicotine products is presented in Table 3. Among all heated tobacco users (n = 44), the levels of interleukin 8 were significantly higher when compared to controls: 6.86 vs. 3.95 (p = 0.01) (Table 3). Among exclusive heated tobacco users (n = 33), the levels of interleukin 8 were also significantly higher when compared to controls: 7.76 vs. 3.95 (p = 0.01) (Table 4). Among all heated tobacco users (n = 44), interleukin 8 level was positively correlated (r = 0.37; p = 0.01) with the daily number of heated tobacco sticks (Table 5). This correlation was not statistically significant in subanalysis for exclusive heated tobacco users (Table 6). There were no statistically significant differences (p > 0.05) in the levels of 9 other blood biomarkers of inflammation, including IL-1β, IL-6, IL-10, IL-12p70, TNF-α, CRP, fibrinogen and adhesion molecules (VCAM-1 and ICAM-1) between heated tobacco users and healthy males who do not use nicotine products (Tables 3, 4, 5, 6).

**Table 3:** Blood biomarkers in regular heated tobacco users (all) and control group.

	Study group		Mean	SD	SE	Median	Min	Max	p
		n							
IL-1β [pg/ml]	H	44	1.91	1.02	0.15	1.60	0.59	5.43	0.9*
	C	48	2.07	1.34	0.19	1.73	0.29	7.83	
IL-6 [pg/ml]	H	44	2.83	1.12	0.17	2.73	0.97	6.63	0.2*
	C	48	3.57	3.03	0.44	3.11	1.03	21.9	
IL-8 [pg/ml]	H	44	6.86	16.5	2.49	4.37	1.97	113.76	0.01*
	C	48	3.95	0.90	0.13	3.82	2.28	6.66	
IL-10 [pg/ml]	H	44	2.04	0.89	0.13	1.97	0.66	4.52	0.5*
	C	48	2.29	1.21	0.17	1.91	0.66	5.20	
IL-12p70 [pg/ml]	H	44	17.47	11.42	1.72	14.42	0.00	49.76	0.1*
	C	48	13.41	7.72	1.11	12.53	0.55	33.93	
TNF-α [pg/ml]	H	44	13.45	2.98	0.44	13.37	7.73	18.43	0.8*
	C	48	13.57	3.84	0.55	13.23	6.79	26.91	
CRP [mg/l]	H	44	8.26	15.67	2.36	2.20	0.00	89.00	0.2*
	C	48	17.77	59.19	8.54	4.21	0.00	404.03	
Fibrinogen [mg/dl]	H	44	162.86	88.40	13.33	125.20	83.47	358.51	0.2*
	C	48	195.64	99.65	14.38	137.28	86.69	387.80	
VCAM-1 [ng/ml]	H	44	967.43	254.34	38.34	954.85	496.65	1685.09	0.9**
	C	48	959.28	265.51	38.32	920.52	460.61	1482.51	
ICAM-1 [ng/ml]	H	44	394.54	448.81	67.66	250.27	98.25	2458.535	0.7*
	C	48	424.29	485.77	70.11	236.11	27.48	1999.79	

*SD* standard deviation, *SE* standard error, *Min* minimum, *Max* maximum, *H* heated tobacco users, *C* controls.

*U Mann–Whitney test; **Student’s *t*-test.

Significant values are in bold.

**Table 4:** Blood biomarkers in exclusive regular heated tobacco users and control group.

	Study group		Mean	SD	SE	Median	Min	Max	p
		n							
IL-1β [pg/ml]	He	33	1.95	1.11	0.19	1.56	0.59	5.43	0.9*
	C	48	2.07	1.34	0.19	1.73	0.29	7.83	
IL-6 [pg/ml]	He	33	2.96	1.18	0.21	2.78	0.97	6.63	0.4*
	C	48	3.57	3.03	0.44	3.11	1.03	21.9	
IL-8 [pg/ml]	He	33	7.76	19.06	3.32	4.39	2.33	113.76	0.01*
	C	48	3.95	0.90	0.13	3.82	2.28	6.66	
IL-10 [pg/ml]	He	33	2.05	0.86	0.15	2.02	0.91	4.51	0.6*
	C	48	2.29	1.21	0.17	1.91	0.66	5.20	
IL-12p70 [pg/ml]	He	33	17.77	12.5	2.18	14.53	0.00	49.76	0.2*
	C	48	13.41	7.72	1.11	12.53	0.55	33.93	
TNF-α [pg/ml]	He	33	12.78	2.73	0.48	12.33	7.73	18.44	0.5*
	C	48	13.57	3.84	0.55	13.23	6.79	26.91	
CRP [mg/l]	He	33	8.22	16.67	2.90	1.96	0.00	89.00	0.1*
	C	48	17.77	59.19	8.54	4.21	0.00	404.03	
Fibrinogen [mg/dl]	He	33	161.80	88.97	15.49	124.89	83.47	358.51	0.2*
	C	48	195.64	99.65	14.38	137.28	86.69	387.80	
VCAM-1 [ng/ml]	He	33	902.31	215.96	37.59	906.31	496.65	1384.20	0.3**
	C	48	959.28	265.51	38.32	920.52	460.61	1482.51	
ICAM-1 [ng/ml]	He	33	366.67	358.52	62.41	248.84	98.25	1743.55	0.9*
	C	48	424.29	485.77	70.11	236.11	27.48	1999.79	

*SD* standard deviation, *SE* standard error, *Min* minimum, *Max* maximum, *He* exclusive heated tobacco users, *C* controls.

*U Mann–Whitney test; **Student’s t-test.

Significant values are in bold.

**Table 5:** Correlation between the levels of blood biomarkers and the daily number of heated tobacco sticks used in the last 90 days among all heated tobacco users (n = 44).

	r	p
IL-1β & daily number of heated tobacco sticks	− 0.11	0.5
IL-6 & daily number of heated tobacco sticks	− 0.01	0.9
IL-8 & daily number of heated tobacco sticks	0.37	**0.01**
IL-10 & daily number of heated tobacco sticks	− 0.02	0.9
IL-12p70 & daily number of heated tobacco sticks	0.11	0.46
TNF-α & daily number of heated tobacco sticks	0.03	0.83
CRP & daily number of heated tobacco sticks	− 0.11	0.5
Fibrinogen & daily number of heated tobacco sticks	− 0.09	0.5
VCAM-1 & daily number of heated tobacco sticks	− 0.27	0.08
ICAM-1 & daily number of heated tobacco sticks	− 0.20	0.2

*r* the Spearman rank correlation coefficient.

Significant values are in bold.

**Table 6:** Correlation between the levels of blood biomarkers and the daily number of heated tobacco sticks used in the last 90 days among exclusive heated tobacco users (n = 33).

	r	p
IL-1β & daily number of heated tobacco sticks	− 0.04	0.8
IL-6 & daily number of heated tobacco sticks	− 0.10	0.6
IL-8 & daily number of heated tobacco sticks	0.08	0.6
IL-10 & daily number of heated tobacco sticks	− 0.20	0.3
IL-12p70 & daily number of heated tobacco sticks	0.03	0.9
TNF-α & daily number of heated tobacco sticks	− 0.06	0.8
CRP & daily number of heated tobacco sticks	0.01	0.9
Fibrinogen & daily number of heated tobacco sticks	− 0.17	0.3
VCAM-1 & daily number of heated tobacco sticks	− 0.18	0.3
ICAM-1 & daily number of heated tobacco sticks	− 0.11	0.5

*r* the Spearman rank correlation coefficient.

## Discussion

This is one of the first human-based case–control studies on the blood biomarkers of inflammation of regular (past 90 days) heated tobacco use. Out of 10 blood biomarkers analyzed in this study, only interleukin 8 levels were significantly (p < 0.05) higher among heated tobacco users compared to healthy males who do not use nicotine products. These differences were observed both among all heated tobacco users (exclusive and dual users with cigarettes or e-cigarettes) and exclusive heated tobacco users. The level of IL-8 raised with the daily number of heated tobacco sticks consumed by HTP users.

Most of the currently available data on the health effects of heated tobacco use are limited to laboratory-based experimental studies or studies on short-term health effects of HTP use among humans^3,9,10,14–17^. Previously published human-based studies assessed the levels of different biomarkers immediately (mostly within 5 min) after the use of heated tobacco sticks^15–17^. Small airway obstruction and resistance as well as a decrease in fractional exhaled nitric oxide were reported as the immediate health effects of HTP use^15,16^. Moreover, an increase in heart rate and blood pressure, increased arterial stiffness, and platelet thrombus formation were described as cardiovascular effects of heated tobacco use^17^. Findings from experimental studies on cell lines and animal models suggest that HTP use may evoke chronic inflammation^14,21–23^. Based on mice models, Sawa et al. reported that short-term exposure to HTP aerosols may increase oxidative stress and induce the secretion of inflammatory cytokines: IL-6 and GM-CSF^14^. Using mice models, Gu et al. reported that chronic exposure to aerosol generated by HTP use resulted in impaired pulmonary function and lung tissue damage^21^. Bhat et al. reported that HTP use induces inflammatory immune-cell accumulation in the lungs as well as augments the levels of proinflammatory cytokines and chemokines in the BAL fluid^22^. Yamamoto et al. reported that HTP use induced IL-8 overexpression with an increase in human monocyte THP-1 cell lines^23^.

In this study, the past 90 days of use of heated tobacco products evoked a significant increase in IL-8 levels, when compared to healthy adults who do not use nicotine products. The levels of IL-8 in blood samples collected from HTP users were elevated regardless of the pattern of heated tobacco use – exclusive HTP use or dual-use (HTPs and cigarettes or e-cigarettes). Interleukin 8 (IL-8) is a major mediator of inflammation and acts as a chemoattractant for neutrophils, basophils, and T cells^24,25^. Previous studies showed that cigarette smoke induces IL-8 release from macrophages. IL-8 is a key chemokine during the initiation and progression of tobacco-related lung inflammation as well as the development of chronic obstructive pulmonary disease (COPD)^26,27^. Findings from cancer research^25^ also showed that elevated levels of IL-8 correspond to increased severity of numerous cancers, including melanoma^28^. breast^29^, renal^30^, prostate^31^ gastric^32^, and colorectal cancers^33^. Our findings suggest that chronic use of heated tobacco products may lead to the development of lung diseases (especially COPD) and cancer progression.

It is estimated that over half of HTP users use heated tobacco with other nicotine products, mostly cigarettes or e-cigarettes (so-called dual users)^34^. In this study, elevated levels of IL-8 were observed also among exclusive heated tobacco users, which suggests that heated tobacco use is the sufficient factor that increases the IL-8 levels. Further studies should analyze differences in blood biomarkers of inflammation between exclusive heated tobacco users and dual users.

Among all heated tobacco users, interleukin 8 level was positively correlated with the daily number of heated tobacco sticks, which suggests a dose-dependent reaction. This observation is in line with the previous findings from tobacco research which proved that the risk of diseases is dose-dependent and varies on the number of cigarettes smoked per day^35,36^.

Heated tobacco products are marketed as less harmful alternatives to cigarettes^3,11^. Findings from the toxicological studies suggest that HTPs compared to cigarettes may be products with a reduced risk of respiratory diseases, cardiovascular diseases, and cancer^2,9^. Out of 10 different biomarkers analyzed in this study, the level of only one biomarker (IL-8) was significantly higher among HTP users compared to controls. There were no differences in the levels of important biomarkers like TNF-α, CRP, fibrinogen, and adhesion molecules as well as other interleukins. In this study, exclusive cigarette smokers were not included as a separate group, and the blood biomarkers of inflammation were compared between heated tobacco users and nicotine-free controls. IL-6 is a predictor of the frequency of COPD exacerbation^37^ and is linked to chronic inflammation-related cancers^38^. However, in this study, there were no statistically significant differences in IL-6 levels between HTP users and the control group. C-reactive protein levels are raised in stable COPD patients^39^. In this study, the mean age of the subjects was 33 years and participants diagnosed with COPD were excluded, so changes in CRP observed usually in patients with COPD were not observed. There were no significant differences in VCAM-1 and ICAM-1, biomarkers associated with cardiovascular disease risk^40^, but due to the younger age of the study population, this observation should be analyzed carefully as Lyytinen et al.^17^ revealed potential cardiovascular risk of HTP use. The mode of operation of heated tobacco products differs from cigarette smoking, so the impact of regular heated tobacco use on biomarkers of inflammation may differ from those observed in cigarette smokers. However, due to the limited sample size (preliminary results), our findings should be interpreted carefully and point to the need for further cohort studies and prospective analyses.

This study has practical implications for tobacco control and public health. Our findings suggest that regular heated tobacco use may lead to the development of chronic diseases (especially respiratory diseases) and the risk is dose dependent. However, the risk of diseases may be lower than among cigarette smokers. Further cohort studies are needed to assess the impact of regular heated tobacco use on the risk of chronic diseases, especially those evoked by chronic inflammation.

There are several limitations of this study. This is a single-point case–control study. The history of heated tobacco use was based on self-reported data but verified by the cotinine levels and set of questions on tobacco use patterns. Findings are based on single-point observations, and follow-ups are needed to assess the impact of heated tobacco use on the dynamics of blood biomarkers (especially proinflammatory cytokines) changes. This is a pilot study, so the number of participants is low. The follow-up study is planned for 2024 and the more advanced analyses will be published after the completion of the study group. In one subject, the IL-8 levels were 113.76 pg/ml, however, even after the exclusions of this subject from the analysis, differences in IL-8 levels between heated tobacco users and control were statistically significant. This study aimed to assess the real-life pattern of heated tobacco use, so exposure to HTPs differs between the participants. Nevertheless, this is one of the first case–control studies on the health effects of heated tobacco use, carried out in humans. Due to the increasing prevalence of HTP use and limited scientific data on their health consequences, investigating cause-and-effect relationships in this area is a new challenge for public health. The results of our study demonstrated that only one of the ten blood-based biomarker of inflammation (IL-8) was significantly higher among the effect use of heated tobacco on blood-based biomarkers of inflammation and higher interleukin 8, one of the main measures of inflammation, in users of heated tobacco compared to non-smokers. Given the lack of significant changes in the other biomarkers of inflammation, the relevance of increases in IL8 needs further investigations to better understand the complex interplay among various interleukins.

In conclusion, daily use of heated tobacco during the last 90 days was associated with an increased level of IL-8 among heated tobacco users compared to healthy controls who do not use nicotine products. An increase in IL-8 level was positively correlated with the number of heated tobacco sticks consumed per day. There were no differences in 9 of 10 blood biomarkers of inflammation between HTP users and controls, so heated tobacco products may be a modified risk tobacco product compared to combustible cigarettes.

## Data Availability

The datasets generated during and analysed during the current study are available from the corresponding author on reasonable request.
